# Optimizing clustering of CDR3 sequences using natural language processing, Word2Vec, and KMeans

**DOI:** 10.3389/fbinf.2025.1623488

**Published:** 2025-10-02

**Authors:** Sanskriti Baranwal, Ricardo Avila Sanchez, Clement-Andi Edet, Erick Chastain, Inimary Toby

**Affiliations:** 1 University of Dallas, Computer Science Department, Irving, TX, United States; 2 University of Dallas, Biology Department, Irving, TX, United States

**Keywords:** acute respiratory disease syndrome (ARDS, ), BioNLP, bioinformatics & computational biology, Word2vec, unsupervised learning

## Abstract

T-cell receptor (TCR) sequencing has emerged as a powerful tool for understanding adaptive immune responses, yet challenges persist in deciphering the immense diversity of Complementarity-Determining Region 3 (CDR3) sequences. This study presents a novel natural language processing (NLP)-based pipeline to cluster CDR3 sequences from TCR β-chain repertoires using Word2Vec embeddings, principal component analysis (PCA), and KMeans clustering. Focusing on Acute Respiratory Distress Syndrome (ARDS), a life-threatening inflammatory lung condition, we trained Word2Vec models on healthy controls and applied unsupervised clustering across ARDS, non-ARDS, and control datasets. Dimensionality-reduced embeddings revealed clear distinctions in repertoire structure: control samples exhibited tight, low-diversity clusters; ARDS patients showed high dispersion and numerous diffuse clusters indicative of repertoire disruption; and non-ARDS samples displayed intermediate organization. These differences suggest that immune activation states are embedded in the structural topology of the CDR3 space. Our framework successfully captured these latent patterns, offering a scalable approach to biomarker discovery. This study not only reinforces the utility of NLP in immunological analysis but also paves the way for data-driven immune monitoring in critical care and personalized diagnostics.

## Introduction

Acute respiratory distress syndrome (ARDS) is a life-threatening condition affecting nearly 190,000 individuals annually in the United States and nearly 10% of ICU patients globally, with high morbidity and mortality rates despite advances in supportive care (Bellani et al., 2016; Matthay et al., 2019). Clinically, ARDS is characterized by acute onset respiratory failure, bilateral infiltrates on chest imaging, and hypoxemia not fully explained by cardiac failure or fluid overload (Sharma and Toney, 2023). Its heterogeneous etiology, ranging from pneumonia and sepsis to aspiration and trauma, complicates therapeutic strategies (Fan et al., 2018). Although ventilatory support remains the cornerstone of ARDS management, the lack of targeted therapies underscores the need to better understand its pathophysiology (Ma et al., 2025).

Recent studies have begun to highlight the immunological underpinnings of ARDS, with particular attention to the role of T cells in mediating both lung injury and repair (Hey et al., 2023). Repertoire-level analysis of T-cell receptors (TCRs), especially the β-chain complementarity-determining region 3 (CDR3β), offers a window into the adaptive immune response in ARDS (Matthay et al., 2023). The CDR3 region plays a central role in antigen specificity due to its high variability generated through V(D)J recombination (Reilly et al., 2018). This recombination process creates an enormous diversity of TCRs, enabling recognition of a vast array of antigens (Cave et al., 2024). Previous work has shown that profiling this diversity can reveal clonal expansion and antigen-driven responses in diseases such as cancer and viral infections (Wick et al., 2024; Glanville et al., 2017).

Traditional alignment-based tools often fail to fully capture the nuanced similarities in TCR sequences, particularly for short and hypervariable regions like CDR3β (Dash et al., 2017). These limitations have prompted the adoption of natural language processing (NLP) techniques for biological sequence analysis. In particular, Word2Vec, a method originally developed for human language modeling, has been successfully applied to biological sequences, generating dense vector representations that preserve contextual relationships between amino acids (Emerson et al., 2017; Robins et al., 2009). When applied to immunological data, such representations have shown promise in identifying motifs and discriminating between immune states (Shugay et al., 2014; Mikolov et al., 2013).

Combining these embeddings with dimensionality reduction methods like principal component analysis (PCA) and clustering techniques such as KMeans has enabled more interpretable visualizations and subgroup discovery within immune repertoires (Heinzinger et al., 2019; Zhang et al., 2023). This approach has been applied in models such as DeepTCR, which uses deep learning to capture complex structural patterns in TCRs (Park et al., 2023), and TCRMatch, a tool that enables high-throughput similarity searches based on CDR3 content (Bolotin et al., 2015). While these frameworks have shown efficacy in oncology (Hou et al., 2021), infectious disease settings (Laing et al., 2020), and autoimmune profiling (Isacchini et al., 2024), their application in ARDS remains limited.

In this study, we utilize Word2Vec and KMeans to analyze CDR3β sequences derived from ARDS patients, non-ARDS ICU controls, and healthy individuals. By embedding and clustering sequences, we aim to identify structural shifts and repertoire disorganization indicative of disease state. Our approach builds on prior work in computational immunology (Larman et al., 2011; Vig et al., 2021), TCR repertoire analysis (Wolock and Klein, 2022; Katayama et al., 2022), and machine learning for immunoprofiling (Textor et al., 2023; Sidhom et al., 2021), while addressing the pressing need for scalable and interpretable models in ARDS research (Mazzotti et al., 2022; Chronister et al., 2021; Alley et al., 2019).

## Methods

### Data acquisition

TCRβ immune sequencing data were obtained in FASTA format following high-throughput sequencing of genomic DNA extracted from lung fluid samples, as described in our previous work (Hey et al., 2023). Genomic DNA had been extracted using the Qiagen miniprep genomic DNA kit and submitted to Adaptive Biotechnologies for TCRβ profiling using their validated ImmunoSEQ® platform. This process yields annotated immune repertoire datasets with high accuracy and reproducibility, incorporating built-in controls to correct for PCR bias and ensure quantitative integrity. The resulting datasets included CDR3 sequence information, V(D)J gene segment assignments, and repertoire metrics across all samples. Additionally, healthy control samples were concurrently extracted from NCBI for use in the study (Supplementary Tables S8–S10).

### Sequence annotation using IgBlast

For downstream analysis, raw TCRβ sequences in FASTA format were processed using NCBI’s IgBlast tool to annotate V, D, and J gene usage and to extract the complementarity-determining region 3 (CDR3). IgBlast identifies CDR3 boundaries using conserved motifs, specifically a cysteine (C) residue at the start and a phenylalanine (F) or glycine (G) residue at the end of the region, consistent with established immunogenetic annotation criteria. The “Analyze T-cell receptor (TR) sequences” option was selected within the IgBlast interface, and sequences were uploaded for automated processing. Following alignment and annotation, output files were reviewed, and the clonotype summary tables were exported for further curation (Supplementary Tables S11–S17).

To ensure analytical focus on biologically functional sequences, the output was filtered to retain only productive rearrangements. Non-productive or incomplete rearrangements were excluded. The curated clonotype data were subsequently converted into CSV format and used for downstream applications, including embedding, clustering, and comparative analyses of CDR3 features across ARDS and non-ARDS cohorts.

### Data preprocessing

A Python script was developed to preprocess the cleaned TCRB sequence data by extracting the CDR3 amino acid sequences along with their corresponding V-gene annotations. Although the V-gene metadata was retained, it was not used directly in the clustering pipeline but preserved for potential future applications such as stratified analysis or supervised learning. The output of this preprocessing step was saved as CSV files, each containing only the relevant CDR3 sequences and V-gene information. The same IgBlast annotation and filtering procedures were consistently applied to all datasets, including the healthy control group. However, in contrast to the ARDS patient data, all healthy control sequences were merged into a single comprehensive dataset to provide a broader and more diverse representation of non-disease immune repertoire. This merged dataset was used to train the Word2Vec model, ensuring that the resulting embeddings captured generalizable patterns across healthy individuals. While the Word2Vec model was trained on a pooled dataset of healthy control samples to maximize generalizability and capture broad semantic relationships between CDR3 motifs, all downstream analyses, including clustering, PCA, Levene’s test, and dispersion measurements, were conducted on unpooled individual samples. This separation ensures that ARDS, non-ARDS were analyzed under equivalent statistical assumptions, avoiding confounding effects introduced by sample aggregation. Our comparisons are therefore made between like units (individual repertoires), preserving the integrity of group-level inferences.

### Word2Vec model training

The Word2Vec model was trained on the merged healthy control dataset. During training, CDR3 sequences were converted into high-dimensional vector representations that captured semantic and relational information between sequences. Each CDR3 amino acid sequence was first segmented into overlapping 3-mers (trigrams) using a sliding window approach. This approach enabled local context capture similar to linguistic tokenization techniques in NLP. The Word2Vec model was implemented using the Gensim library (v4.3) with the following hyperparameters: vector size = 100, window size = 5, minimum token count = 1, skip-gram architecture (sg = 1), and trained over 10 epochs. These parameters were empirically selected to balance embedding granularity with interpretability and clustering performance (Wolock and Klein, 2022; Chronister et al., 2021).

The final dataset consisted of 254 healthy control sequences, 139 ARDS sequences, and 115 non-ARDS sequences (total = 508). This yielded a Word2Vec vocabulary of 20 unique 3-mers. With an average CDR3 length of 15 amino acids, the corpus contained over 7,000 total tokens, though redundancy and the restricted amino acid alphabet limited the number of distinct trigrams. Using a vector size of 100 and accounting for both input and output embeddings, the model contained approximately 4,000 trainable parameters (calculated as 2 × vector size × vocabulary size). While the vocabulary size may appear small compared to typical NLP corpora, this is expected for TCR CDR3 sequences due to the finite amino acid alphabet and the biologically constrained motif space; in this setting, even a limited trigram set can capture meaningful immunological patterns.

### Clustering and statistical analysis

Dimensionality reduction was first applied using Principal Component Analysis (PCA). The Silhouette Coefficient was utilized to determine the optimal number of principal components (L) to retain. PCA was then used to reduce the dimensionality of the Word2Vec embeddings while preserving the underlying structure of the data. A total of 20 principal components were retained based on an explained variance threshold of 90%.

To identify the optimal number of clusters (k) for the KMeans algorithm, silhouette coefficients were calculated for k values ranging from 2 to 15. The ARDS dataset achieved its highest silhouette coefficient at k = 9 (∼0.42), and the non-ARDS dataset at k = 2 (0.60). The healthy control dataset has a maximum of its silhouette coefficient at k = 2, but this was considered a trivial partition, so we instead used the elbow method to choose k = 5 (which has a silhouette coefficient of ∼0.345). These results correspond to the PCA cluster visualizations in Figures 2–4 and are further supported by the elbow and silhouette plots provided in Supplementary Figures S1–S6. The k values for ARDS and non-ARDS were based on the silhouette coefficient and that of the healthy control dataset was based on the elbow plot (the maximum silhouette coefficient gave a trivial result for the latter case, due to the silhouette coefficient favoring large clusters). Although the silhouette score for ARDS is lower, this is consistent with the diffuse, overlapping clusters observed in ARDS repertoires and reflects true biological heterogeneity rather than methodological limitations. Together, these findings indicate varying levels of repertoire diversity and immune activation across clinical groups. Based on these optimal k values, KMeans clustering was applied to the vectorized CDR3 sequence embeddings for each cohort. To ensure robustness and mitigate overfitting, K-Fold Cross-Validation was employed, repeatedly splitting the data into training and validation sets.

### Visualization of clustering results

The clustering results from KMeans were visualized to interpret the distribution and organization of TCRB sequences among ARDS patients and healthy controls. Comparative analyses were conducted to examine differences in clustering patterns between the two groups. This methodological approach, integrating immune sequencing, machine learning, and natural language processing, allowed for an in-depth examination of immune repertoire variations. The combination of IgBlast-based sequence annotation, Word2Vec embeddings, PCA, and KMeans clustering provided a robust framework for uncovering immune response patterns, with the use of cross-validation and clustering evaluation metrics further enhancing the reliability and generalizability of the findings.

## Results

**FIGURE 1:**
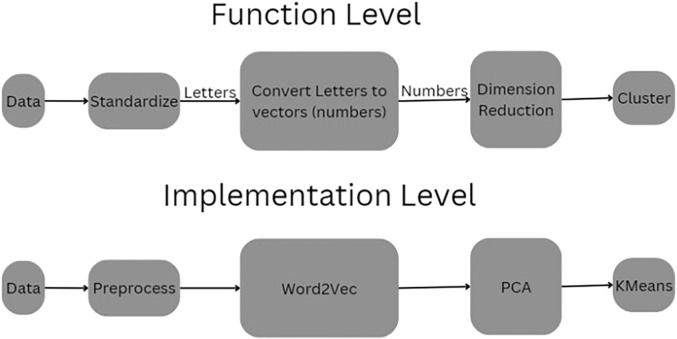
Pipeline of the Process. This schematic illustrates the dual-layer structure of the CDR3 sequence clustering pipeline. The Function Level (top) abstracts the process conceptually, beginning with raw sequence data that is first standardized, then transformed from amino acid letters into numerical vectors. These vectors undergo dimensionality reduction before being grouped into clusters. The Implementation Level (bottom) presents the actual computational tools used: raw data is preprocessed and passed through a Word2Vec embedding model, followed by Principal Component Analysis (PCA) for dimensionality reduction and KMeans for unsupervised clustering.

The methodological pipeline of this study across both conceptual and implementation layers is shown in Figure 1. At the functional level, raw immunosequencing data undergoes a transformation pipeline beginning with standardization, followed by semantic vectorization of CDR3 amino acid sequences using natural language processing (NLP) techniques. These sequences, originally represented as strings of characters, are embedded as numerical vectors to capture underlying relationships and biological patterns. These vectors are then subjected to dimensionality reduction—an essential step to mitigate the curse of dimensionality and facilitate downstream clustering. The final step involves unsupervised clustering to identify distinct immune subpopulations. The implementation-level diagram concretizes this process through specific tools: preprocessing scripts extract and clean the CDR3 sequences, Word2Vec is used to generate embeddings (Mikolov et al., 2013), principal component analysis (PCA) is employed to reduce dimensionality, and KMeans is applied for cluster assignment. Together, this dual-layer schematic provides both a conceptual abstraction and a transparent, reproducible computational workflow.

To statistically compare the spread of clusters between groups, we applied Levene’s test for equality of variances on the mean Euclidean distances between CDR3 embeddings and their cluster centroids (Supplementary Figure S7) between groups. The analysis revealed that ARDS samples had significantly greater intra-cluster dispersion compared to non-ARDS samples (p = 1.66 × 10^−6^). These results quantitatively support the hypothesis that ARDS is associated with heightened immune perturbation and repertoire fragmentation.

**FIGURE 2:**
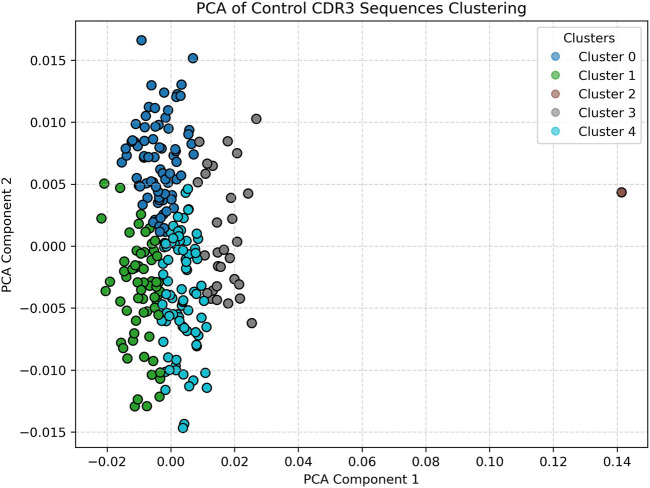
Healthy (Control) Data clustered visual. Displays tightly packed and vertically aligned clusters, indicating a relatively stable and conserved T-cell receptor repertoire. The low dispersion and well-defined clusters reflect immune homeostasis. Optimal clustering for controls was determined as k = 5 using the elbow method.

PCA visualization of clustered CDR3 sequence embeddings from a representative healthy control sample is shown in Figure 2. The dimensionality-reduced embeddings form clearly demarcated clusters, with relatively low intra-cluster variance and tight spatial grouping along the first two principal components. The concentration of points and compactness of clusters suggest a stable and relatively conserved T-cell repertoire structure in immunologically homeostatic individuals. The presence of fewer, well-defined clusters may reflect baseline V-gene usage patterns and low antigenic pressure, consistent with findings in non-pathogenic states (Glanville et al., 2017; Emerson et al., 2017). Notably, the separation between clusters indicates that the embedding space, informed by Word2Vec, effectively captures semantically meaningful differences among CDR3 sequences even in control conditions (Heinzinger et al., 2019; Zhang et al., 2023).

**FIGURE 3:**
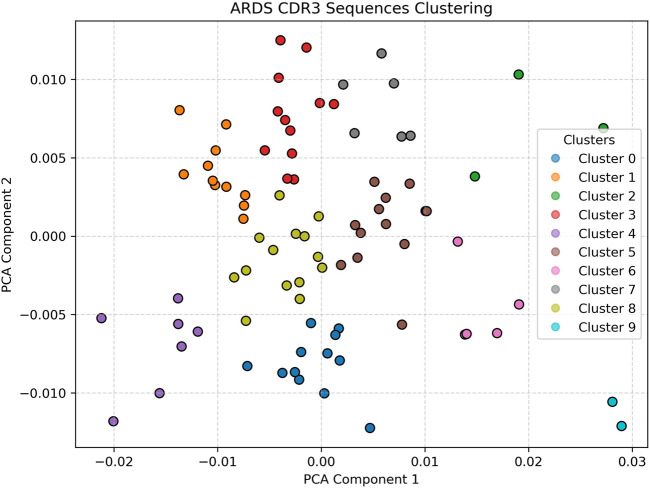
ARDS Patient clustering visualization Shows greater cluster spread, higher heterogeneity, and increased number of clusters, reflecting repertoire disruption. The diffuse and overlapping clusters suggest heightened T-cell activation and clonal diversification due to systemic inflammation. Silhouette analysis identified k = 9 as the optimal cluster number for ARDS samples.

In contrast, Figure 3 displays the PCA projection of CDR3 sequences derived from a representative ARDS sample. Here, the embedding space reveals a pronounced increase in both the number of clusters and their spatial dispersion. The clusters are more diffuse and demonstrate overlapping boundaries, a hallmark of repertoire perturbation under inflammatory stress. This fragmentation and diversification of the CDR3 space likely reflects heightened T-cell activity and clonal expansion in response to the systemic inflammation characteristic of acute respiratory distress syndrome (Matthay et al., 2019; Fan et al., 2018; Hey et al., 2023). The emergence of multiple novel clusters in this sample, which are not observed in the healthy control, underscores the potential of this approach for identifying ARDS-specific immune signatures. The increased heterogeneity may also suggest a breakdown in repertoire regularity, pointing to dysregulated T-cell dynamics under critical illness (Matthay et al., 2023; Reilly et al., 2018).

**FIGURE 4:**
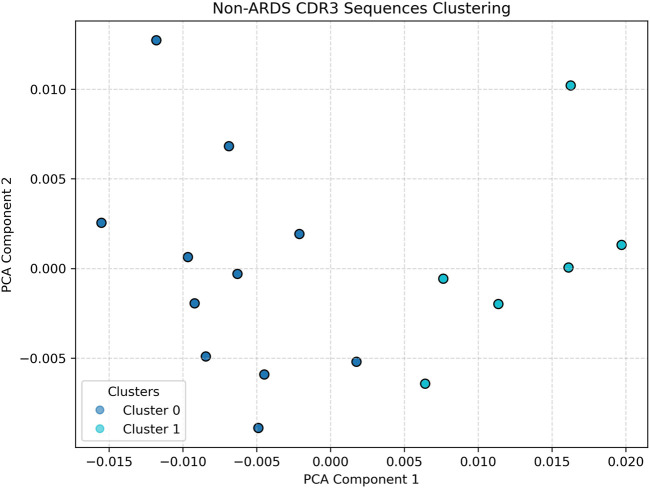
Non-ARDS Patient clustering visualization. Illustrates an intermediate profile. Cluster structures are more defined than in the ARDS sample, suggesting partial immune activation or chronic inflammation without the full dysregulation seen in ARDS. Silhouette analysis identified k = 2 as optimal for non-ARDS samples.

Compared to the respective ARDS sample, the CDR3 embeddings in Figure 4, taken from a representative non-ARDS sample, exhibit moderate diversity, with clusters that are more defined and spatially constrained, though still more dispersed than in the control group. This intermediate pattern likely reflects partial immune activation without the full spectrum of systemic immune dysregulation observed in ARDS. The presence of a limited number of distinct clusters could be indicative of a targeted T-cell response, potentially tied to pathogen-specific recognition or chronic inflammation, rather than a broad-based, polyclonal expansion (Sharma and Toney, 2023; Cave et al., 2024; Wick et al., 2024). This sample provides a valuable comparative case, demonstrating that not all lung pathologies induce the same degree of immune repertoire disruption.

Taken together, our results illustrate the capacity of the Word2Vec–PCA–KMeans pipeline to resolve fine-grained distinctions in immune repertoire structure across clinical phenotypes (Hey et al., 2023; Robins et al., 2009; Park et al., 2023). Control datasets yield tightly clustered and uniform embeddings, indicative of repertoire homeostasis. ARDS samples from our study demonstrated significant repertoire diversification and immune disruption, while non-ARDS samples occupied a middle ground in both cluster spread and heterogeneity. These findings support our hypothesis that unsupervised clustering of CDR3 embeddings could reveal disease-specific immunological fingerprints, offering potential for non-invasive biomarker development and immune monitoring in critical care settings.

## Discussion

This study demonstrates that unsupervised machine learning can meaningfully cluster CDR3 sequences from T-cell receptors, revealing distinct immune repertoire structures in ARDS patients compared to healthy individuals. By combining Word2Vec embeddings with PCA and KMeans clustering, we observed stark differences in repertoire topology. The ARDS group exhibited the highest number of clusters (k = 9), suggesting a more fragmented or polyclonal response, whereas the non-ARDS group had the fewest clusters (k = 2), pointing toward repertoire contraction or clonal dominance. Control samples were optimally clustered at k = 5 (based on the elbow method), reflecting a balanced and conserved immune state consistent with repertoire homeostasis (Hey et al., 2023; Glanville et al., 2017).

To reinforce these findings, we incorporated quantitative validation via silhouette analysis and the elbow method (Supplementary Figures S1–S6), which confirmed optimal clustering structures across phenotypes, and further demonstrated increased dispersion and fragmentation in ARDS samples. In particular, the silhouette coefficients showed maxima at the k values used, with the elbow plots included for comparison (showing different optima). The silhouette coefficients confirmed the chosen k-values for the ARDS and non-ARDS group, with the highest scores for non-ARDS at k = 2 (0.60) and ARDS at k = 9 (∼0.42). As the silhouette coefficient is a more principled measure for finding the number of clusters, we used it *in lieu* of the elbow plot when the former does not give trivial results (as mentioned before). Levene’s test additionally revealed significantly greater intra-cluster variance in ARDS compared to both control and non-ARDS samples, quantitatively supporting our hypothesis of repertoire disorganization in severe disease.This difference in optimal clustering granularity highlights the variation in immune activation and organization across conditions. Levene’s test further supported this interpretation by demonstrating significantly greater intra-cluster variance in ARDS samples compared to controls, indicative of clonal skewing or repertoire narrowing. It is important to note that while control sequences were merged for embedding model training, all clustering and statistical comparisons in this study were performed using unpooled, individual datasets. All clustering and statistical comparisons were performed using unpooled datasets to preserve inter-individual variability and group comparability (Rosati et al., 2017).

These results support our hypothesis that ARDS is associated with T-cell repertoire diversification and disorganization, aligning with prior studies that observed immune dysregulation in ARDS and sepsis (Matthay et al., 2019; Fan et al., 2018; Hey et al., 2023). Other work also supports the implication of adaptive immune responses, particularly T-cell activity, not only in lung injury but also in resolution and repair processes in ARDS (Ma et al., 2025; Matthay et al., 2023; Wick et al., 2024). The structural patterns we observed in CDR3 embeddings extend these findings by offering a computationally tractable representation of immune variation beyond clonality or V-gene usage (Emerson et al., 2017; Robins et al., 2009; Shugay et al., 2014). This approach provides a powerful alternative to conventional alignment-based techniques like MiXCR or sequence similarity metrics that are often sensitive to mutation and sampling noise (Bolotin et al., 2015; Rosati et al., 2017). Word2Vec’s ability to encode contextual similarity allows it to capture motifs and structural features within immune repertoires that may not be evident through traditional metrics (Mikolov et al., 2013; Heinzinger et al., 2019). Our findings are in line with recent efforts to apply embedding models to TCRs in the context of infectious diseases and cancer, including DeepTCR (Sidhom et al., 2021), Immune2vec (Wolock and Klein, 2022), and TCRMatch (Chronister et al., 2021).

In addition, this work resonates with observations from recent COVID-19 immune profiling studies, which show that disease severity can be reflected in changes in repertoire structure and clonal expansion (Hou et al., 2021; Laing et al., 2020). We are also in the process of employing the techniques described here to the examination of other lung pathologies. Similar repertoire remodeling in ARDS, especially the increase in cluster dispersion, may indicate heightened T-cell activation, clonal exhaustion, or bystander activation during systemic inflammation (Fan et al., 2018; Wick et al., 2024; Isacchini et al., 2024). Our study adds to this narrative by showing that embedding-based clustering can quantitatively distinguish these states, offering potential for non-invasive immune monitoring (Hey et al., 2023; Park et al., 2023; Textor et al., 2023).

In addition to this, our study presents opportunities for further exploration. First, we relied exclusively on Word2Vec embeddings, which capture local amino acid context but may miss long-range or structural features critical to TCR function (Heinzinger et al., 2019; Zhang et al., 2023; Vig et al., 2021). While we used Word2Vec for its simplicity and interpretability, we chose it over newer transformer-based models such as ProtBERT and ProtT5 for several reasons. First, Word2Vec produces embeddings that are intuitive and biologically interpretable, allowing us to link local amino acid context with immune repertoire structure. This interpretability is especially valuable for unsupervised clustering, where black-box features from larger models may hinder biological insight.

Second, Word2Vec is computationally efficient and robust on smaller datasets, which is crucial for projects like ours where data is limited. In contrast, ProtBERT and ProtT5 require large-scale data and extensive GPU resources, and may be prone to overfitting when applied to niche domains such as lung-specific TCR repertoires.

Third, as demonstrated in prior work (Wolock and Klein, 2022; Sidhom et al., 2021; Chronister et al., 2021), Word2Vec embeddings have proven effective in capturing immunologically relevant motifs, particularly in short, variable sequences like CDR3s. Its trigram tokenization mirrors biologically meaningful substructures and has been shown to preserve important biochemical relationships, unlike transformer models trained primarily on full-length proteins.

In future work, we plan to benchmark against ProtBERT and state-of-the-art models like Mamba (2024) to determine whether their increased complexity yields measurable benefits in downstream performance. For this study, however, Word2Vec offered the most pragmatic and transparent solution.

We also plan to integrate clinical metadata—such as ARDS severity scores, etiology, treatment outcomes, and cytokine panels—into future analyses. This would enable clustering patterns to be correlated with patient trajectories and therapeutic responses, potentially enabling prognostic modeling (Reilly et al., 2018; ElSayed et al., 2023; Greiff et al., 2017). Incorporating other omics data, such as transcriptomics or cytokine profiles, may also improve repertoire interpretation and provide a more holistic view of immune function during critical illness (Cave et al., 2024; Emerson et al., 2017).

Importantly, our findings suggest that immune repertoires encode latent, quantifiable structure reflective of disease state. That structure, once identified, can be used to guide biomarker discovery, therapeutic development, and personalized diagnostics. Embedding-based clustering pipelines such as ours are efficient, interpretable, and scalable, providing a powerful lens through which to understand complex immunological phenomena (Larman et al., 2011; Alley et al., 2019; Sethna et al., 2020). In conclusion, this study presents a compelling use case for NLP-inspired tools in the study of immune repertoires in ARDS. By translating amino acid sequences into vector space and applying unsupervised clustering, we revealed meaningful differences between disease and control samples. These findings lay the groundwork for future immune-monitoring pipelines in critical care settings and offer a blueprint for applying machine learning to immunological data across diseases.

## Data Availability

The original contributions presented in the study are included in the article/Supplementary Material, further inquiries can be directed to the corresponding author.
