# Supplementary figures and images for: Optimizing clustering of CDR3 sequences using natural language processing, Word2Vec, and KMeans
Source: Front Bioinform. 2025 Oct 2;5:1623488. doi: 10.3389/fbinf.2025.1623488 (PMC12528129; doi:10.3389/fbinf.2025.1623488)

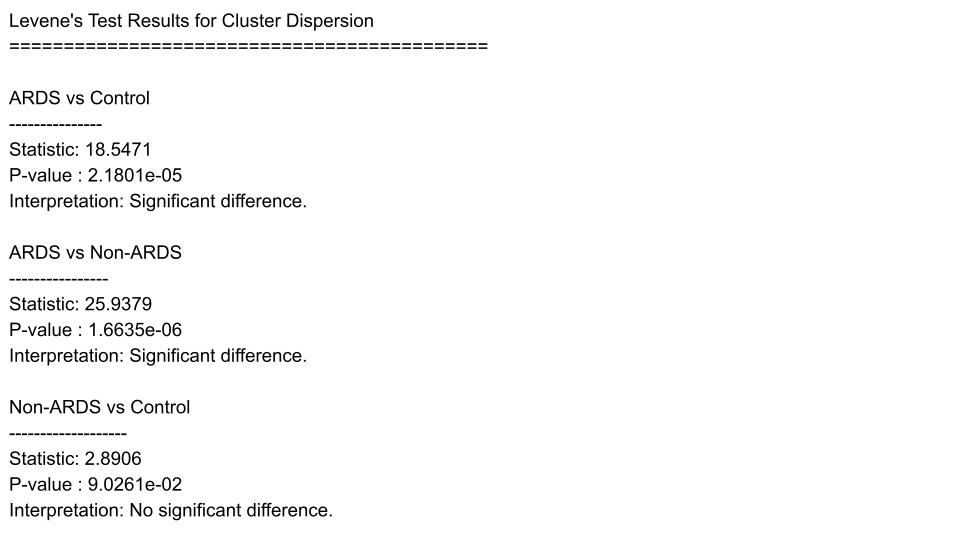

Supplement: Supplementary file 4 [file Image7.jpeg]

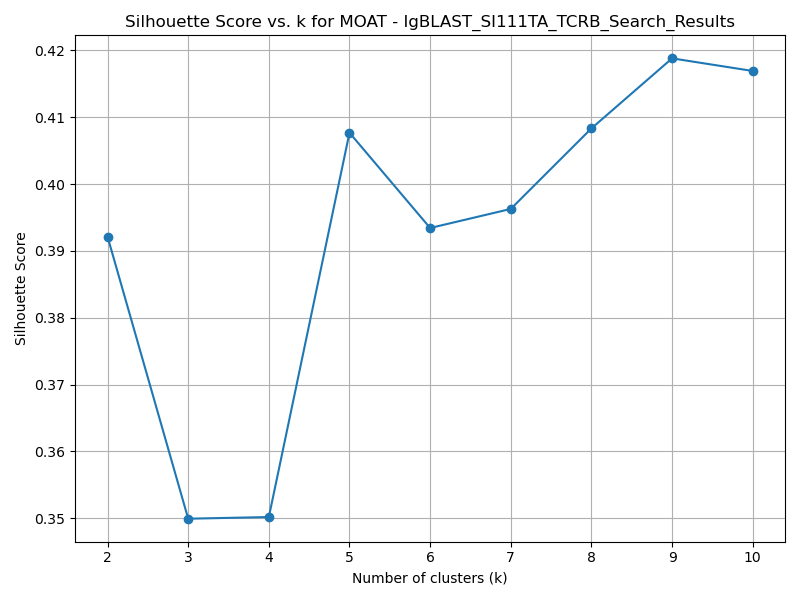

Supplement: Supplementary file 8 [file Image4.png]

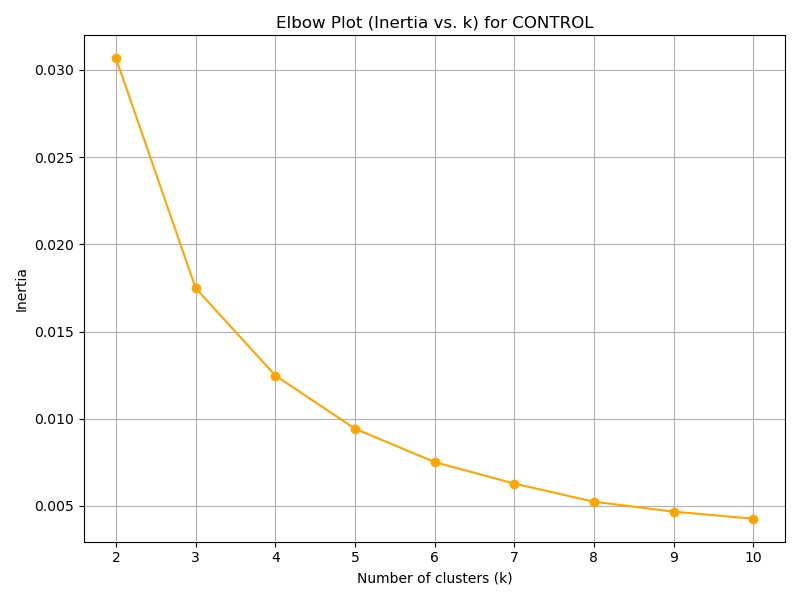

Supplement: Supplementary file 10 [file Image2.png]

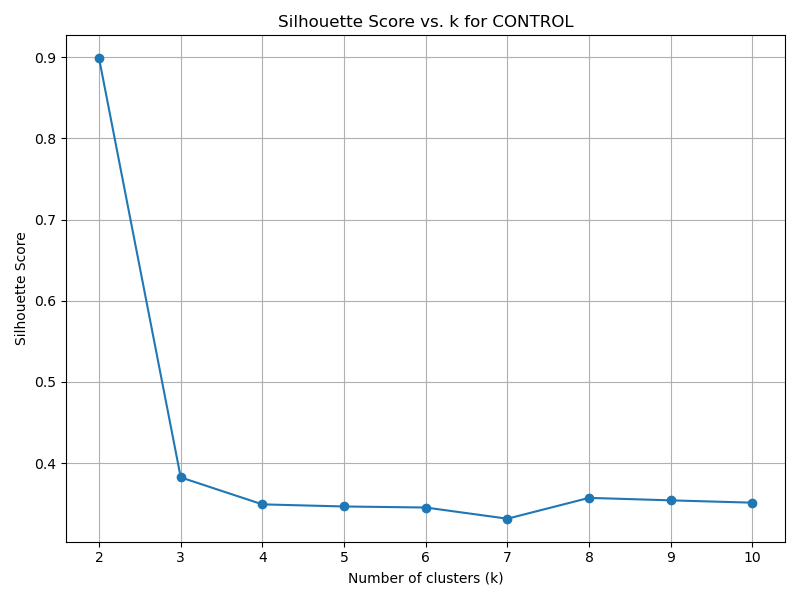

Supplement: Supplementary file 11 [file Image1.png]

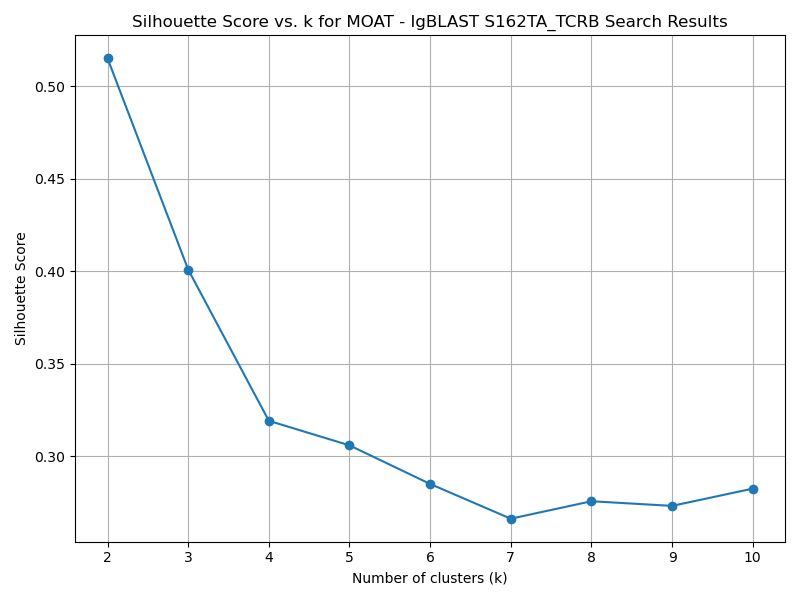

Supplement: Supplementary file 13 [file Image6.png]

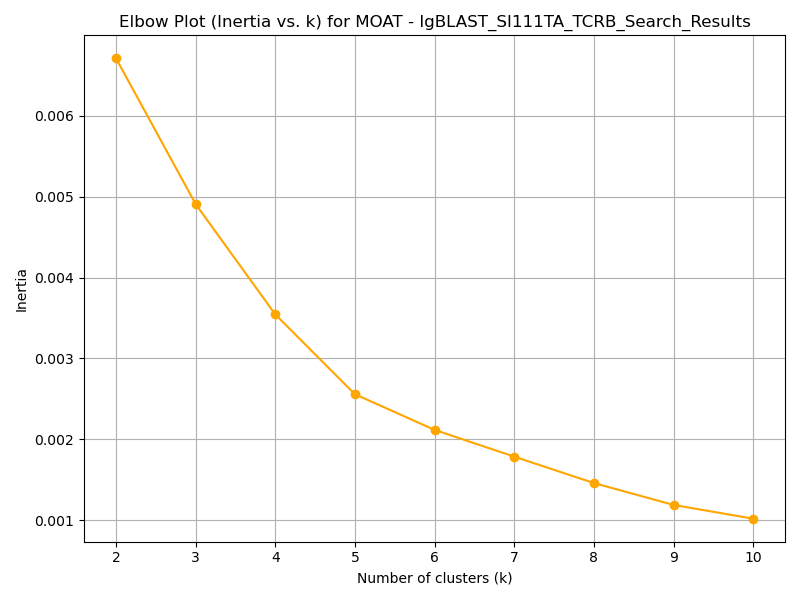

Supplement: Supplementary file 14 [file Image3.png]
